# Curcumin Modulates DNA Methylation in Colorectal Cancer Cells

**DOI:** 10.1371/journal.pone.0057709

**Published:** 2013-02-27

**Authors:** Alexander Link, Francesc Balaguer, Yan Shen, Juan Jose Lozano, Hon-Chiu E. Leung, C. Richard Boland, Ajay Goel

**Affiliations:** 1 Gastrointestinal Cancer Research Laboratory, Division of Gastroenterology, Baylor Research Institute, Baylor University Medical Center, Dallas, Texas, United States of America; 2 Department of Gastroenterology, Hepatology and Infectious Diseases, Otto-von-Guericke University, Magdeburg, Germany; 3 Department of Gastroenterology, Hospital Clínic, Centro de Investigación Biomédica en Red de Enfermedades Hepáticas y Digestivas (CIBEREHD), IDIBAPS, University of Barcelona, Barcelona, Spain; 4 Dan L Duncan Cancer Center, Baylor College of Medicine, Houston, Texas, United States of America; Peking University Health Science Center, China

## Abstract

**Aim:**

Recent evidence suggests that several dietary polyphenols may exert their chemopreventive effect through epigenetic modifications. Curcumin is one of the most widely studied dietary chemopreventive agents for colon cancer prevention, however, its effects on epigenetic alterations, particularly DNA methylation, remain unclear. Using systematic genome-wide approaches, we aimed to elucidate the effect of curcumin on DNA methylation alterations in colorectal cancer cells.

**Materials and Methods:**

To evaluate the effect of curcumin on DNA methylation, three CRC cell lines, HCT116, HT29 and RKO, were treated with curcumin. 5-aza-2′-deoxycytidine (5-aza-CdR) and trichostatin A treated cells were used as positive and negative controls for DNA methylation changes, respectively. Methylation status of LINE-1 repeat elements, DNA promoter methylation microarrays and gene expression arrays were used to assess global methylation and gene expression changes. Validation was performed using independent microarrays, quantitative bisulfite pyrosequencing, and qPCR.

**Results:**

As expected, genome-wide methylation microarrays revealed significant DNA hypomethylation in 5-aza-CdR-treated cells (mean β-values of 0.12), however, non-significant changes in mean β-values were observed in curcumin-treated cells. In comparison to mock-treated cells, curcumin-induced DNA methylation alterations occurred in a time-dependent manner. In contrast to the generalized, non-specific global hypomethylation observed with 5-aza-CdR, curcumin treatment resulted in methylation changes at selected, partially-methylated loci, instead of fully-methylated CpG sites. DNA methylation alterations were supported by corresponding changes in gene expression at both up- and down-regulated genes in various CRC cell lines.

**Conclusions:**

Our data provide previously unrecognized evidence for curcumin-mediated DNA methylation alterations as a potential mechanism of colon cancer chemoprevention. In contrast to non-specific global hypomethylation induced by 5-aza-CdR, curcumin-induced methylation changes occurred only in a subset of partially-methylated genes, which provides additional mechanistic insights into the potent chemopreventive effect of this dietary nutraceutical.

## Introduction

Colorectal cancer (CRC) is one of the leading causes of death worldwide, being responsible for approximately 10% of total cancer-related mortality [1]. About 3–5% of all CRCs are due to inherited genetic defects and up to 25% of patients may have some degree of familiality for this disease, but the majority of CRCs occur in a sporadic manner in the absence of a documented family history. Increasing evidence indicates that in addition to genetic instability phenotypes such as chromosomal and microsatellite instability, epigenetic alterations that include DNA methylation alterations, histone modifications and alterations in miRNA expression, may play an important role in the initiation and progression of CRC [2]–[4].

In contrast to genetic defects, epigenetic alterations are more dynamic and can be influenced by aging, environmental, lifestyle and dietary factors, which are believed to play a major role in the development of over two-thirds of all human cancers [5]–[7]. Aberrant DNA methylation consisting of both focal *hypermethylation* of promoter CpG islands that results in the transcriptional silencing of genes, and global *hypomethylation* of DNA that facilitates chromosomal instability and aneuploidy, and is one of the most extensively studied epigenetic events in cancer [8]–[11]. Furthermore, given that DNA methylation alterations are potentially reversible, and often precede genetic events during multistep colorectal carcinogenesis, provide an exciting and promising opportunity for cancer prevention and treatment [12]–[17].

Based upon this concept, epigenetic therapy is currently being explored, with the goal of preventing cancer cells from acquiring aberrant DNA methylation and helping to restore ‘normal’ DNA methylation and gene expression patterns to crucial cancer-related genes. Consequently, nucleoside analogues such as 5-azacytidine and 5-aza-2′-deoxycytidine (5-aza-CdR) have been identified as potent DNA methyl transferase (DNMT) inhibitors. Incorporation of these nucleoside analogues directly into DNA during replication as well as proteosomal degradation of the DNMT1 enzyme that catalyzes *de novo* hypermethylation constitutes two key mechanisms that are responsible for their demethylating activities [18]–[20]. There is a growing realization that reversal of aberrant DNA methylation patterns in cancer cells may be effective for its treatment and prevention [21]. Several such nucleoside analogues are currently being explored for the treatment of hematological malignancies and are in early-stage clinical trials for other solid malignancies; unfortunately, the broad usefulness of these agents has been hampered by toxicity and adverse side effects. Moreover, the non-specificity that permits global hypomethylation of even fully methylated genes and results in chromosomal instability limits the use of these compounds as potential chemopreventive agents [8], [10]. In a quest to seek alternative chemopreventive strategies, several phytochemicals have emerged as potentially potent choices by virtue of a lack of significant toxicity profiles [22]. Furthermore, data suggest that perturbations in dietary nutrients such as folate and selenium can affect DNA methylation, both *in vitro* and *in vivo*, as a result of an inhibition of DNMT1 protein expression and enzymatic activity [23]. Dietary polyphenols, such as (−)-epigallocatechin-3-gallate (EGCG) from green tea and genistein from soybean, have also been shown to inhibit DNMT activity in cancer cell lines [24], [25]. DNMT inhibitory activity is associated with demethylation and reactivation of several methylation-silenced genes such as *p16, RARβ, MGMT, MLH1, BTG3* and *GSTP1* in human cancer cells, suggesting a possible chemopreventive effect due to epigenetic modification induced by these dietary botanicals [25], [26]. However, the variable demethylating efficacy of polyphenols remains poorly understood because most published studies have reported data for only handful of genes, and many of these epigenetic effects have not been reproducible in independent studies [27], [28].

Curcumin (diferuloylmethane), a natural compound derived from the spice turmeric (*Curcuma longa*), has been used for treatment of various inflammatory diseases in traditional Indian and Chinese systems of medicine. In recent decades, an extensive and in-depth body of published scientific literature has revealed that the chemopreventive effects of curcumin are mediated by a variety of molecular mechanisms, including its direct or indirect interaction with various transcription factors, enzymes and regulatory proteins that play a central role in key cancer-related processes such as inflammation, proliferation, survival, migration, angiogenesis, invasion and metastasis [22]. Evidence for the effectiveness of curcumin as an anticancer or chemopreventive agent has been drawn from hundreds of studies undertaken in cell culture systems, animal models and human subjects [29]. More recent studies have begun to recognize curcumin’s effect in modulating epigenetic processes in cancer cells, in which curcumin has been shown to be a histone acetyltransferase (HAT) inhibitor [30], [31], as well as a potential DNMT1 inhibitor, which results in hypomethylation of various genes including RARβ2 in cervical cancer cells [32]–[34]. However, the role of curcumin as a DNA hypomethylating compound has not been systematically evaluated as of yet, and its demethylating potential has not been successfully reproduced in its entirety in other studies [32], [33].

Accordingly, in the present study, we performed a comprehensive and systematic analysis to investigate the effect of curcumin on DNA methylation in colon cancer cells. Genome-wide DNA methylation analyses and simultaneous gene expression profiling studies were performed in CRC cell lines treated with short- and long-term curcumin treatment. Herein, we demonstrate that curcumin modulates DNA methylation alterations in cancer cells; furthermore, such changes are frequently corroborated with corresponding changes in gene expression. Finally, we provide novel evidence, that in contrast to global hypomethylation induced by 5-aza-CdR, DNA methylation changes associated with curcumin treatment occur only in a subset of primarily partially-methylated genes, and in a time dependent manner.

## Materials and Methods

### Cell Culture

Three human CRC cell lines, HCT116 (microsatellite unstable or MSI), RKO (CpG Island methylation phenotype or CIMP) and HT29 (microsatellite stable or MSS), were obtained from the American Type Culture Collection (ATCC, Manassas, VA). Cells were cultured in IMDM medium (Invitrogen, Rockville MD) under standard conditions with 10% fetal bovine serum and 5% CO_2_ at 37°C. The cell line authenticity was frequently confirmed by analyzing various genetic and epigenetic markers every 6–8 months.

### Curcumin Treatment

Curcumin (Sigma-Aldrich, MO, US) stocks were prepared by dissolving it in dimethylsulphoxide (DMSO) at 10 mM, and small aliquots were stored at −20°C until use. To determine the effect of curcumin on DNA methylation, all three CRC cell lines were exposed to 7.5–10 µM curcumin for short-term (6 days; STC) or long-term (240 days; LTC) treatment. Fresh curcumin-containing culture medium was replaced every second day during the course of treatment. Control cell lines without curcumin treatment were grown with each set of treatments for the same duration.

### 5-aza-CdR and TSA Treatment

5-aza-CdR treatment was performed as described previously [35]. Briefly, 5-aza-CdR was dissolved in PBS (pH 7.5) at 5 mM and small aliquots were kept frozen at −20°C. Twenty four hours after seeding, CRC cells were treated with 2.5 µM 5-aza-CdR (Sigma-Aldrich, MO, US) for 24 h, and the cells were harvested after 48 h. Trichostatin A (TSA) was dissolved in ethanol at 0.3 µM. Cells were seeded equally and after overnight growth were treated with TSA for 24 h. Cells were pelleted and stored at −80°C until analysis.

### MTT Viability Assay

The effects of curcumin on cell viability were determined in an MTT assay, which is based upon 3-(4,5-dimethylthiazol-2-yl)-2,5-diphenyltetrazolium bromide uptake. Cells (2×10^3^/well) were seeded in 96-well plates 24 h prior to curcumin treatment. After 72 h of curcumin treatment, MTT solution was added to each well and incubated for 2 h at 37°C. Cells were incubated with SDS Buffer (10%) with 0.01 M HCl overnight and the absorbance was measured at 570 nm using a spectrophotometer. Independent experiments were performed three times in triplicate.

### Bromodeoxyuridine (BrdU) Proliferation Assay

Cell proliferation analyses were performed using the colorimetric Cell Proliferation ELISA, BrdU kit (Roche Diagnostics, Indianapolis, IN) following manufacturer’s instructions. Briefly, 2×10^3^ cells were cultured in 96-well plates and treated with curcumin for 72 h. Proliferation index was measured by BrdU incorporation following manufacturer’s instructions. Experiments were performed in triplicate in 3 independent experiments.

### Plating Efficiency

To test for plating efficiency, cells were plated at a low density in 6-well plates and treated using the treatment protocol described above for 12–16 days until individual cells formed distinctly visible colonies. Thereafter cells were fixed with 10% formalin and stained with 0.1% crystal violet. After washing, plates were air-dried and digital images were taken. Independent experiments were performed three times in triplicate.

### DNA Extraction, Bisulfite Modification and Methylation Profiling (Infinium Methylation Assay)

DNA extraction was performed with the DNeasy Blood & Tissue kit (Qiagen, Valencia, CA) according to manufacturer’s instructions. Genomic DNA was bisulfite modified (EZ DNA methylation Gold Kit, Zymo Research, please add city/state) as described previously [35]. To analyze the effect of curcumin on DNA methylation, we performed DNA methylation profiling using the Infinium methylation assay with HumanMethylation27 BeadChip microarrays (Illumina, San Diego, CA), which are capable of simultaneously analyzing the methylation status of 27,578 individual CpG sites covering over 14,000 genes [36]. Whole genome amplification, labeling, hybridization and scanning were performed according to the manufacturer’s instruction at the genomics core facility at the Dan L. Duncan Cancer Center, Baylor College of Medicine, Houston, TX. Methylation status was measured as the ratio of signal from a methylated probe relative to both methylated and unmethylated probe signals. Methylation ratios were extracted using the Methylation Modules in the Illumina Bead Studio following average normalization, and the quantitative β-value ranged from 0 (0% methylation) to 1 (100% methylation). The p-value cut-off for detectable probe signals was set at 0.05. For the methylation analyses, a Δβ-value of ≥0.1 (10% methylation) was defined as significant [37].

### Quantitative Methylation Analyses

We validated methylation microarray data by independent single gene/promoter quantitative methylation assays. Depending on the methylation assay design features, we used either bisulfite pyrosequencing (PSQ HS 96A pyrosequencing system, Qiagen, Valencia, CA) or real-time based qMSP for quantitative methylation analyses as described previously [37], [38]. Primer sequences used for both methods are provided in **Supplementary Table S1**.

### Gene Expression Microarray Analyses

In parallel with methylation profiling, we performed microarray gene expression analyses following the manufacturer’s instructions and a previously established protocol [39]. Total RNA was isolated using the RNeasy mini-kit (QIAGEN, Valencia, CA) and amplified using Illumina’s TotalPrep RNA Amplification Kit. RNA integrity was assessed using the Agilent 2100 Bioanalyzer. Labeled cRNA was hybridized overnight to Human HT12 V3 chips, washed, and scanned on an Illumina BeadStation-500. Illumina’s BeadStudio version 3.1 was used to generate signal intensity values from the scans, subtract background, and scale each microarray to the median average intensity for all samples (per-chip normalization). Normalization was done using quantiles with the Lumi R-package. Fold-changes were calculated with respect to their respective controls as described elsewhere [35]. Ingenuity pathway analysis (IPA, Ingenuity Systems, Inc. CA, USA) was performed to evaluate und categorize differentially expressed genes into various functional pathways.

### Statistical Analyses

All data were analyzed using Graph Pad Prism 4.0 (San Diego, CA, USA) statistical software. Differences between two groups were analyzed using Student’s t-test. Differences between more than two groups were analyzed using repeated measures ANOVA and Bonferroni’s multiple comparisons as a *post hoc* test. Two sided p-values of <0.05 were regarded significant.

## Results

### Curcumin Inhibits Cell Viability and Proliferation in CRC Cell Lines

**Figure 1:**
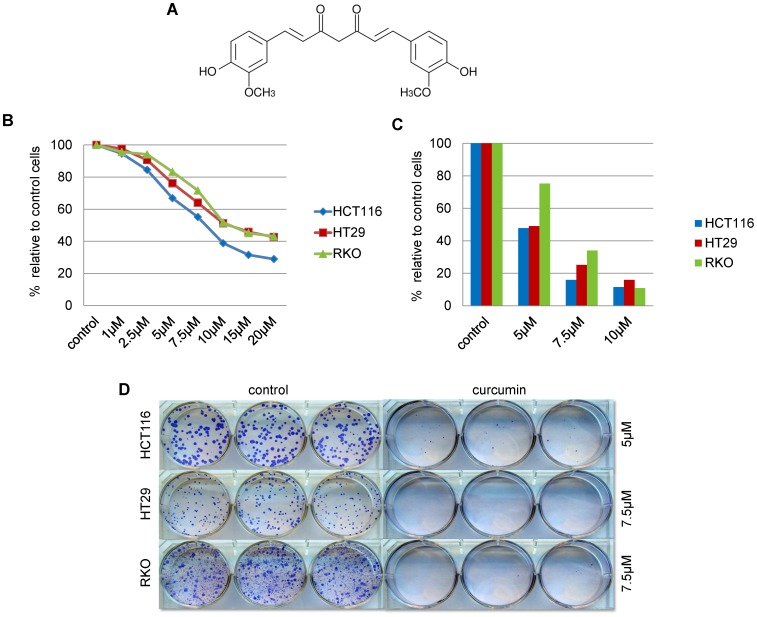
Curcumin inhibits cell viability, proliferation and colony formation of CRC cells. (A) Chemical structure of curcumin: (1*E*,6*E*)-1,7-bis(4-hydroxy-3-methoxyphenyl)-1,6-heptadiene-3,5-dione. (B) MTT and (C) BrdU assays were performed to determine the best effective sub-toxic concentration of curcumin. CRC cells were treated with curcumin at various concentrations for 72 h (data represent the mean of independent experiments performed in triplicate). (D) The long-term anti-proliferative effects were evaluated in a colony formation assay. The cells were treated with curcumin for 12 to 16 days until distinct colonies were visible. Representative images of from an experiment illustrating colony formation results in controls (DMSO treated) and curcumin-treated HCT116, RKO and HT29 cancer cells.

Previous studies have provided multiple levels of evidence for the anti-cancer effects of curcumin by influencing cell proliferation, apoptosis, migration and invasion. To determine the effective and non-toxic curcumin concentrations in our panel of CRC cell lines (**Figure 1A**) we first performed proliferation and cell viability assays in CRC cell lines exposed to various concentrations of this dietary polyphenol. Three human CRC cell lines that represent distinct epigenotypes of human colon cancers were used: HCT116 (microsatellite unstable – MSI - cancer cell line due to germline mutation in the *MLH1,* mismatch repair gene), RKO (MSI cancer cell line associated with *MLH1* promoter hypermethylation in the context of the CpG island promoter methylation phenotype or CIMP) and HT29 (microsatellite stable or MSS, cell line with mutant *KRAS-* and *p53* genes) [40]. MTT and BrdU assays were performed after treatment with either curcumin (0–20 µM) or DMSO for 72 h (**Figure 1B & 1C** **, respectively**). Proliferation of CRC cell lines was exponentially affected in a dose-dependent manner. While concentrations of ≥15 µM were associated with toxicity, the approximate half maximal inhibitory concentrations (IC_50_) of 7.5 µM for HCT116 and 10µM for HT29 and RKO were determined based upon cell proliferation indices. In addition, colony formation assays were also performed over a period of 12–14 days (**Figure 1D**), which further confirmed the optimal dose of 7.5 µM for HCT116, and 10 µM for HT29 and RKO cell lines, for subsequent DNA methylation experiments.

### Curcumin Induces Demethylation of Specific CpG Loci in CRC Cells

**Figure 2:**
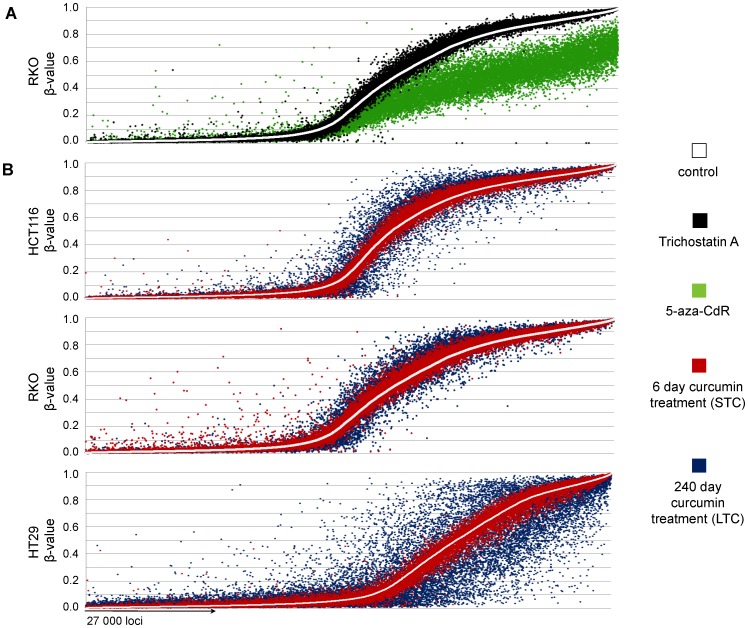
Modification of multiple CpG loci following treatment of CRC cells with curcumin. (A) For the positive control of hypomethylation, we treated RKO cells with 2.5 µM 5-aza-CdR. For the negative control, RKO cells were treated with 0.3 µM trichostatin A (TSA). (B) Three CRC cell lines from different epigenetic phenotypes were treated with effective anti-proliferative concentrations of curcumin (HCT116 7.5 µM, RKO and HT29 10 µM) for 6 and 240 days. Short treatment was associated with few changes in DNA methylation, while long-term treatment with curcumin resulted in extensive changes in CpG methylation. To compare the direct effect of the treatment on the methylation status Δβ_(βcontrol–βtreatment)_ values were calculated accordingly for each CpG locus. The white line represents the ascending order of methylation of CpG loci in control/parental cell lines. Dots represent the direct comparison of matching CpG loci (>27,500) in treated cells.

To analyze genome-wide methylation alterations associated with curcumin treatment, we used Infinium microarrays with over 27,000 CpG loci. First, we treated RKO (a CIMP positive and highly methylated CRC cell line) with 5-aza-CdR (a DNMT inhibitor) and TSA (a potent HDAC inhibitor) as positive and negative controls, respectively. As shown in **Figure 2A**, a single day of treatment with 2.5 µM 5-aza-CdR resulted in widespread demethylation of thousands of CpG loci in RKO cells. In contrast, TSA treatment had a minimal effect on demethylation of any CpG sites. The mean β-values in RKO cells decreased from 0.39 to 0.26 following treatment with 5-aza-CdR, whereas no changes in β-values were seen with TSA treatment, thus confirming the specificity of genome-wide demethylation of various CpG sites following treatment with a DNMT inhibitor in this experimental condition.

To evaluate the effect of curcumin on DNA methylation we used two different treatment models: First, we adopted a commonly used short-term treatment of CRC cells with curcumin over a period of 6 days [24]. Second, we used long-term curcumin treatment for 240 days, a model that probably better mimics the use of curcumin in a chemopreventative setting in humans, and one that was assumed to better represent curcumin-induced epigenetic alterations in clones of cells that have undergone and maintained CpG demethylation, given curcumin’s lower activity compared to commercially available DNMT inhibitors. To examine the effects of curcumin on CpG methylation (**Figure 2B**), we performed parallel comparisons between short- and long-term curcumin treated CRC cell lines. First, we sorted all 27,000 CpG loci in ascending order of methylation based on the methylation status in parental cell lines (white dots that cumulatively appear as a line in **Figure 2**), while corresponding DNA methylation changes following both short- and long-term curcumin treatments are represented as red and blue dots, respectively. The vertical distances of each dot from the “line depicting results from the control cells” represent the level of either hyper- (above the white line) or hypo-methylation (below the white line). As shown in **Figure 2B** **,** short-term curcumin treatment was associated with relatively minor methylation changes in all 3 cell lines, with the corresponding mean β-value changes as follows; 0.389 vs. 0.393 for HCT116, 0.388 vs. 0.396 for RKO and 0.294 vs. 0.291 for HT29. In contrast, long-term curcumin treatment was associated with more pronounced methylation changes compared to control cells (**Figure 2B**), although the net β-value change was not significantly different (0.399 for HCT116, 0.398 for RKO and 0.275 for HT29).

### Curcumin did not Induce Global DNA Methylation Changes

**Figure 3:**
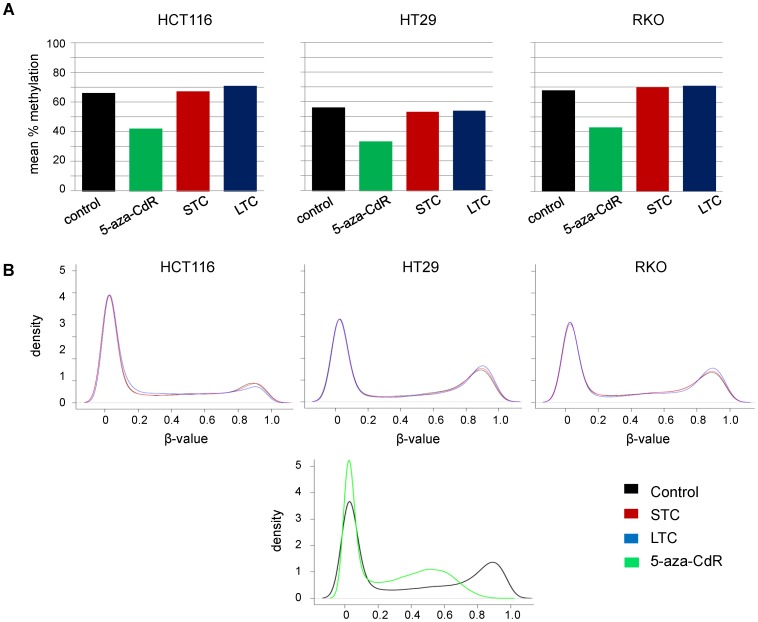
Curcumin treatment is not associated with changes in methylation status of long interspersed nuclear elements-1 (LINE-1) that serve as surrogate markers for global DNA methylation. (A) HCT116, RKO and HT29 were treated with 5-aza-CdR and curcumin and LINE-1 methylation status was determined using bisulfite pyrosequencing. (B) To assess changes in global methylation patterns, density plots were calculated for controls, 5-aza-CdR and curcumin-treated cell lines using Infinium global methylation microarrays. While 5-aza-CdR was responsible for a shift in CpG methylation towards hypomethylation, methylation pattern of the cells after curcumin treatment remained unchanged. Abbreviations: 5-aza-deoxycytidine (5-aza-CdR), short-term curcumin treatment (STC), long treatment with curcumin (LTC).

To evaluate the effect of curcumin on global DNA methylation alterations, we deployed two independent approaches. First, we analyzed the methylation status of LINE-1 elements, which serve as surrogate markers of global methylation, using quantitative bisulfite pyrosequencing [41]. We found that after short- and long-term treatment with curcumin LINE-1 methylation levels were comparable to untreated controls, while those in 5-aza-CdR treated cell lines showed significant decreases in LINE-1 methylation (**Figure 3A**). Second, using the Infinium methylation array data, we visualized global CpG loci density patterns using the density plots. As expected, 5-aza-CdR treatment was associated with a clear shift of the methylation density line towards the unmethylated state, while no obvious changes in global methylation density patterns were found in CRC cell lines treated with curcumin – an observation that is consistent with our LINE-1 methylation results showing an absence of global methylation changes induced by curcumin (**Figure 3B**).

### Validation of Curcumin-induced DNA Methylation Alterations in CRC Cell Lines

**Figure 4:**
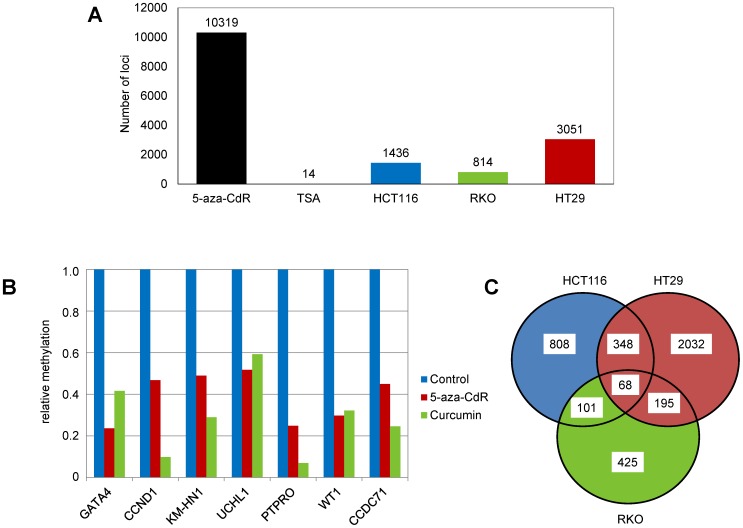
Validation of the curcumin-mediated methylation changes in CRC cell lines. (A) To validate the changes in DNA methylation, the samples were re-analyzed with an Infinium microarray using independently bisulfite modified genomic DNA. The figure represents the number of CpG loci that showed CpG methylation changes with a Δβ-value of ≥0.1 in both experiments. 5-aza-CdR and TSA treated cells were used as positive and negative controls of validation. (B) Quantitative MSP (qMSP) was performed to validate the methylation changes in HCT116. Relative methylation was calculated by normalization of the methylation status of curcumin and 5-aza-CdR treated cells to controls. (C) The Venn diagram shows that CpG methylation changes overlap between HCT116, RKO and HT29 after the treatment with curcumin.

As described above, long-term exposure to curcumin was associated with more significant methylation alterations compared to short-term treated CRC cell lines. Thus, for validation purposes, we narrowed our focus on the effects of long-term curcumin treatment using two different strategies. First, we performed an independent genome-wide methylation microarray analysis (Infinium®) using matched independent bisulfite modified DNA samples from curcumin and control cell lines. As described above, a Δβ-value of 0.1 (equal to 10% methylation) was used as cut-off threshold for differentially methylated loci. **Figure 4A** illustrates the number of differentially methylated loci following long-term curcumin treatment that were shared between both sets of methylation microarrays. Overall we found a high degree of correlation between the two experiments (HCT116-LTC: R^2^ = 0.9669, p<0.0001; RKO-LTC: R^2^ = 0.9508, p<0.0001; HT29-LTC: R^2^ = 0.9089; p<0.0001; RKO 5-aza-CdR: R^2^ = 0.9569; p<0.0001; RKO-TSA: R^2^ = 0.9484; p<0.0001). Accordingly, we confirmed that long-term curcumin treatment was associated with DNA methylation alterations at 1436 loci for HCT116, 814 for RKO and 3051 for HT29 (**Figure 4A**). Second, we successfully validated methylation changes at several randomly selected loci including *KM-HN-1, PTPRO, WT1* and *GATA4,* using a qMSP assay in both curcumin and 5-aza-CdR treated colon cancer cell lines (**Figure 4B**). With the exception of UCHL-1 (β-value of 0–9), all validated loci had β-value between 0.3–0.8.

### Curcumin-induced Methylation Alterations Occur in a gene- and Cell Line-Specific Manner

**Figure 5:**
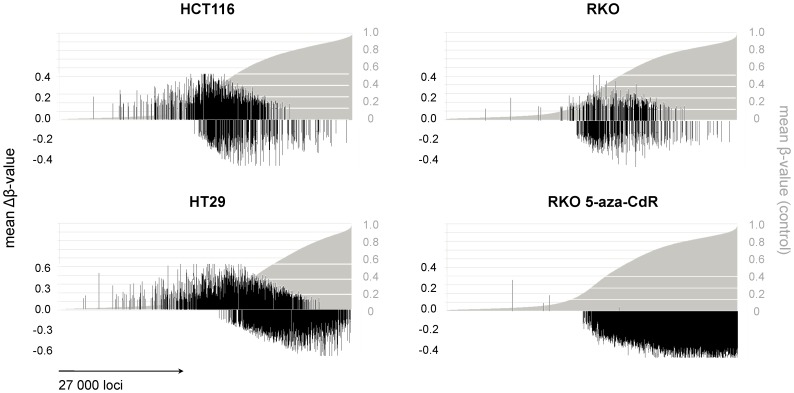
Curcumin-mediated changes in DNA methylation differ among 5-aza-CdR treated cells. The validated CpG loci of the cell lines were ordered in ascending order. The figure represents the magnitude and the location of curcumin-affected CpG loci in relation to control cell lines. The gray curve represents the distribution of the CpG loci in ascending order. The black lines represent the Δβ-values_(βcontrol–βtreatment)_ matching the control cells. The treatment with curcumin was associated with both hyper- and hypomethylation changes, predominantly in partially-methylated CpG loci, while 5-azaCdR treatment was responsible for non-selective hypomethylation.

We next questioned whether curcumin-induced DNA methylation alterations observed in our experiments are gene- or cell line-specific. Taking into account the genetic and epigenetic differences between various CRC cell lines analyzed in this study, we found in one-to-one comparisons that all 3 cell lines shared in up to 30% CpG loci some degree of methylation changes following curcumin treatment. However, we observed that only 68 CpG loci were hypomethylated in all 3 cell lines, suggesting cell line-specific differences in DNA methylation induced by curcumin (**Figure 4C**). Next, we wanted to see whether curcumin-induced methylation alterations are random, or if there is a specific pattern to this epigenetic modification. To answer this question we once again arranged methylation results from all 27,000 CpG sites in ascending order, and overlaid these results with the mean Δβ-value change in curcumin treated cells (**Figure 5**). In addition to both hypo- and hyper-methylation of various CpG sites associated with curcumin treatment, we observed a unique pattern of curcumin-induced methylation changes that clearly differed from the 5-aza-CdR one in all 3 cell lines. As shown on the **Figure 5**, while 5-aza-CdR induced-hypomethylation was detected at all CpG loci, curcumin treatment was associated predominantly with methylation alterations at CpG sites that were partially methylated, an observation that supports a gene-specific demethylation of dynamically methylated CpG loci that are targets of curcumin-induced epigenetic modification.

### Curcumin-induced DNA Methylation Alterations Associate with Corresponding Changes in Gene Expression

**Figure 6:**
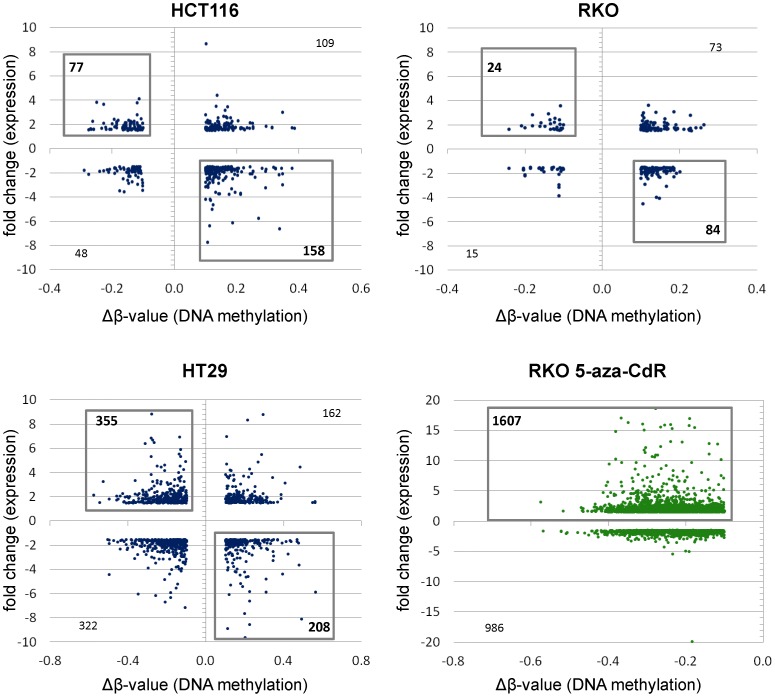
Changes in curcumin-mediated modification of DNA methylation correlate with changes in gene expression. To evaluate if curcumin-mediated changes in DNA methylation correlated with gene expression variation, we performed genome-wide gene expression analyses. Genes that showed reproducible DNA methylation changes after curcumin treatment (Δβ>0.1) were matched with genes that showed ≥1.5-fold differences in gene expression. The black boxes include those genes that showed an inverse correlation between methylation and gene expression changes.

To further understand the biological significance of DNA methylation changes induced by curcumin, we analyzed gene expression profiles in CRC cell lines before and after long-term curcumin treatment. For this analysis, we performed correlative analysis between genome-wide methylation results and the gene expression data. Herein, we selected all CpG loci that had a methylation change of at least 10% (or a Δβ-value ±0.1) and an expression change of at least 1.5-fold between control and curcumin treated cells. Using these criteria, we identified 235 genes in HCT116, 108 genes in RKO and 543 in HT29 cells (**Figure 6**). Ingenuity Pathway Analyses (IPA) revealed that genes with simultaneous DNA methylation and gene expression alterations are frequently involved in multiple important cellular regulatory processes including drug or lipid metabolism, molecular transport, cancer biology, cell signaling, inflammatory response and cell cycle. Detailed categories of such genes in HCT116 cells are presented in **Table 1**. Therefore, our data suggest that methylation changes induced by curcumin have a direct impact on the transcription of various genes that participate in important biological processes that may be instrumental in curcumin-mediated anticancer effects.

**Table 1:** Functional categories of curcumin mediated genes in HCT116 colon cancer cells.

Category	p-value	Molecules
**Drug Metabolism**	2,19E−04–4,38E−02	SULT1A1, ABCC2, NCOR2, SULT1A2, CYP2D6, CES1 (includes EG:1066), SLCO1B1
**Molecular Transport**	2,19E−04–4,38E−02	ABCC2, SLC1A2, PON3, FCGR1A, TRPV6, SLCO2B1, S100A8, SLC25A15, SLCO1B1
**Molecule Biochemistry**	2,19E−04–4,38E−02	SEPP1, FAAH, PON3, PRG3, SLC25A15, SULT1A1, ABCC2, NCOR2, CYP2D6, SULT1A2, SLC1A2, WARS, CES1 (includes EG:1066), PCK1, S100A8, SLCO1B1
**Cancer**	1,07E−03–3,73E−02	AMD1, IER2, CDCA5, AMY2A, CTDSPL, KRT23, GABRB3, AIM2, ECM2, HTATIP2, ITGB8, ERG, PTPN6, WARS, S100A14, REL, WHSC1L1, S100A8, SERPINE1, PTP4A3, FGF12, STARD13, AKAP12, LARGE, SQLE, GPR65, TCL1B, ZNF217, SULT1A2, FGFR3, CES1 (includes EG:1066), DMPK, ZNF23, FGA, HK2, ATP2B2, SLCO1B1, HS3ST2, HPSE, MMP11, NOTCH3, MMP25, QPCT (includes EG:25797), USP9X, IFT57, CALD1, SULT1A1, FSHR, FUT3, CYP2D6, SLC1A2, FZD7, CLC, SDC2, CASP6, PRSS23, NCAM1, PAEP, IL7R, MYOZ2, S100A4, CCND1, SLC2A3, TTC3, ABCC2, NCOR2, OR51E2, DDX19B
**Cell-To-Cell Signaling and Interaction**	2,04E−03–5E−02	HPSE, S100A10, MMRN1, PRG3, GPR56, ITGB8, FUT3, PCDHB4 (includes EG:56131), SLC1A2, CLC, SDC2, TRPV6, S100A8, SERPINE1, NCAM1, IGSF1, PAEP, LGALS9, FCGR1A, UNC119, CHL1, CD1C, FEZ1, GPR176, NCOR2, DRD1, ELF3, LAT2, TSLP, SLC6A5, FGA, PSEN1
**Tissue Development**	2,04E−03–5E−02	NCAM1, IGSF1, LGALS9, HPSE, CCND1, MMRN1, CHL1, ARTN, USP9X, GPR56, ITGB8, FEZ1, FSHR, FUT3, PCDHB4 (includes EG:56131), ELF3, SDC2, TRPV6, FGA, S100A8, SERPINE1, PSEN1
**Gastrointestinal Disease**	2,7E−03–2,7E−03	CDCA5, MYOZ2, STARD13, S100A4, MMP11, SQLE, CCND1, SLC2A3, GPR65, QPCT (includes EG:25797), MMP25, CALD1, FSHR, FUT3, SULT1A2, FZD7, WARS, FGFR3, CLC, PRSS23, ZNF23, PTP4A3, SERPINE1, FGF12
**Inflammatory Response**	2,87E−03–4,38E−02	PAEP, IL7R, FCGR1A, S100A10, GPR65, PRG3, AIM2, CD1C, TCF7, NCOR2, LAT2, TSLP, CLC, TREM1, PRSS23, S100A8
**Hepatic System Disease**	3,15E−03–1,48E−02	SULT1A1, ABCC2, SULT1A2, SLCO1B1
**Inflammatory Disease**	3,15E−03–3,75E−02	ABCC2, PTPN6, SLC1A2, SDC2, SCAP, S100A10, S100A8, TCF7, SLCO1B1
**Gene Expression**	3,5E−03–4,38E−02	TAF11, FSHR, RORA, REL, MAP2K3, LY9, CCND1, HTATIP2
**Cell Cycle**	4,37E−03–4,38E−02	PPAP2C, CAMKK2, S100A4, CHN2, CCND1, VPS18
**Cell Morphology**	5,8E−03–4,51E−02	NCAM1, CEP170, FEZ1, AMD1, FCGR1A, STARD13, LST1, SDC2, CCND1, CASP6, TRPV6, SERPINE1
**Growth and Proliferation**	7,22E−03–4,83E−02	AMD1, PPAP2C, RORA, IL4I1, MMP11, USP9X, ARTN, ITGB8, PTPN6, ERG, FSHR, WARS, REL, SDC2, S100A8, ACP1, GPNMB, SERPINE1, IL7R, UNC119, STARD13, LST1, CHN2, CCND1, NCOR2, ELF3, TSLP, FGFR3, HK2, PSEN1
**Cellular Compromise**	9,1E−03–4,87E−02	FUT3, CSPG4, HPSE, S100A4, TREM1, CASP6, PRSS23, IFT57
**Cell Signaling**	1,31E−02–4,38E−02	DRD1, LAT2, CAMKK2, FCGR1A, TRPV6, SLC25A15, PSEN1
**Metabolic Disease**	1,37E−02–4,38E−02	REG3G, SLC16A6, AMY2A, GABRB3, HSPA1L, HPCAL1, SLC25A15, HTATIP2, BARX2, GPR56, ERG, WARS, ARHGEF11, TSGA10, CYB5A, SERPINE1, FGF12, LST1, LARGE, ZNF615, DRD1, DOPEY2, SLC6A5, GALNT10, HLA-DPA1, PTPRE, FGA, HK2, PSEN1, SLCO1B1, HS3ST2, CHODL, RORA, HMBOX1, STXBP1, TRDN, NCAM1, MYOZ2, IL7R, CHN2, CCND1, SLC2A3, TTC3, CHL1, FAM49A, LONRF2, DPCR1, PCK1, OR51E2
**Cellular Assembly**	1,48E−02–4,51E−02	NCAM1, FEZ1, CSPG4, FCGR1A, LST1, CCND1, CASP6, HK2, CALD1
**Cellular Development**	1,48E−02–4,38E−02	ELF3, IL7R, CCND1, TRPV6, ZNF217, PSEN1
**Cellular Movement**	1,48E−02–4,38E−02	AMD1, CSPG4, HPSE, PTP4A3
**Tumor Morphology**	1,48E−02–4,87E−02	FUT3, HPSE, SDC2, CCND1
**Cell Death**	3,41E−02–4,38E−02	CSPG4, UNC5B, S100A8, PSEN1

HCT116 CRC cells were treated with curcumin for 240 days. Infinium genome-wide methylation analyses and Illumina gene expression analyses were performed to access the curcumin-mediated changes. Matching genes with curcumin-mediated methylation changes of β-value ≥0.1 and genes with ≥1.5-fold difference in gene expression were used for Ingenuity Pathway Analyses.

### Curcumin-mediated Gene Expression Changes are Similar in Short and Long-term Treatments

**Figure 7:**
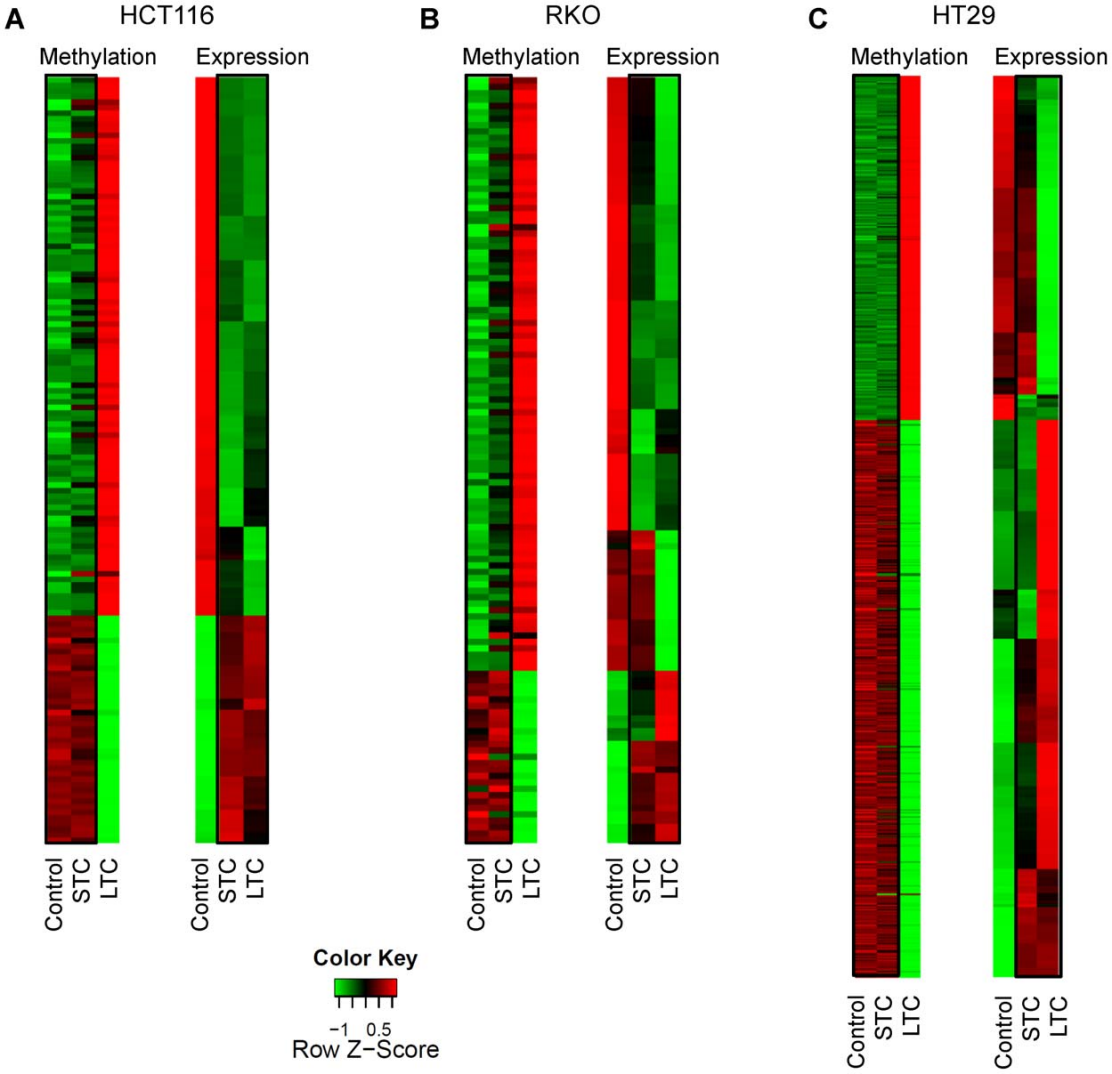
Comparable gene expression changes among short, and long-term curcumin-treated cells. Heatmaps displaying genes with the most discordant expression when comparing gene expression and methylation profiles with HCT116, HT29 and RKO control and treated samples. Genes were selected based on the significance of the inverse correlation between methylation and gene expression as shown in Figure 6. Rows represent genes, and columns represent samples; the intensity of each color denotes the standardized ratio between each value and the average methylation/expression of each gene across all samples. Red pixels correspond to an increased abundance of the transcript in the indicated samples, whereas green pixels indicate decreased transcript levels.

Having shown that curcumin-induced DNA methylation change occur in a gene- and cell specific manner in comparison to 5-aza-CdR, we hypothesized that modification of the CpG methylation following long-term curcumin exposure may, in part, reflect a secondary indirect effect, which results in a stable impact on curcumin-induced changes in gene expression in cancer cells. To address this issue, we compared methylation microarray results and gene expression data from controls and curcumin-treated cells (both short- and long-term). For this purpose, we selected the subsets of genes that showed significant inverse correlation between CpG methylation and gene expression (**Figure 6**) in each CRC cell line following long-term curcumin exposure. These analyses revealed that methylation profiles of short-term curcumin-treated cells were similar to control cells but significantly different from long-term treated cells (**Figure 7**). In contrast, as shown in the heat maps in **Figure 7**, the gene expression profiles in short- and long-term curcumin treated cells were remarkably similar in all 3 CRC cell lines, at least partly supporting our hypothesis that long-term curcumin treatment may influence DNA methylation through the regulation of gene expression. Having demonstrated the concordance for gene expression data between short- and long-term curcumin treated cells, we next questioned if any of these genes are related to the NF-κB pathway, which is effectively inhibited by curcumin. As shown on the **Suppl. Fig S1**, multiple genes were directly related to NF-κB, providing additional evidence for the potential for secondary modification of DNA methylation following gene expression.

## Discussion

In the present study, we have systematically examined the potential of curcumin to modulate DNA methylation and gene expression in CRC cells. Based upon our results, we provide novel evidence that curcumin, a dietary polyphenol from the spice turmeric, is an effective anti-cancer agent that can mediate DNA methylation alterations in CRC cells. More importantly, in contrast to the DNMT inhibitor 5-aza-CdR, curcumin did not induce widespread non-specific global methylation changes, but rather affected only a subset of specific genes, supporting the concept that its methylation-modulatory activity is perhaps an indirect phenomenon that is independent of DNMT inhibitory activity. Our data also demonstrate that curcumin is associated with DNA methylation changes at predominantly partially-methylated genes, indicative of a less potent, but perhaps biologically relevant DNA demethylating potential. Our observation of a significant correlation between DNA methylation and gene expression changes further highlighted the biological significance of curcumin-induced DNA methylation alterations in CRC cells.

Curcumin is a potent antitumor compound that has been shown to influence multiple gene targets in various cancer-related pathways [22]. Although recent experimental and epidemiological studies have provided new evidence for curcumin-induced histone modifications [15], data regarding its ability to modulate DNA methylation in cancer cells is relatively scarce [33], [34]. This study is the first of its kind in this regard, in which we have comprehensively and systematically investigated the effect of curcumin on DNA methylation alterations in various CRC cell lines. To overcome the potential limitation of target-based approaches in previous studies [27], [28], we performed an unbiased genome-wide methylation microarray analysis, which, to our best knowledge has not been used for this type of analyses before. To better appreciate the effect of curcumin on this epigenetic change, we also included both positive (5-aza-CdR) and negative (TSA) controls for assay validation. Furthermore, we analyzed a panel of 3 CRC cells lines representative of heterogeneous epigenetic phenotypes in order to overcome the potential biological variability or bias, and increase the reliability of our results. Using independent methods such as DNA methylation arrays, qMSP and bisulfite pyrosequencing, we provide evidence that curcumin indeed modulates gene-specific DNA methylation, but does not influence global methylation patterns in CRC cells. These results contrast with Liu et al., who used theoretical molecular docking studies and ex-vivo experiments to demonstrate that curcumin inhibited the activity of *M.sssl* and induced global hypomethylation in MV4-11 leukemia cells [34]. Likewise, Liu et al. and Kuck et al. used molecular docking screening studies to suggest that curcumin might interact with DNMT1 [33], [34]. Although there may be multiple biological explanations for the differences observed between our data and these previous publications (including differences in cell lines, duration of treatment, choice of methods etc.), the methodological differences are most critical in explaining the disparity in results [27], [28]. While our experiments were systematically performed in cultured CRC cell lines using a wide variety of microarray and validation approaches, Liu et al. simply performed either theoretical or ex-vivo studies on genomic DNA treated with various concentrations of curcumin, potentially limiting the biological interpretability of their preliminary findings [33], [34].

It has been suggested that multiple polyphenols such as EGCG, genistein and lycopene may induce methylation changes in a time-dependent manner [15], and accordingly, their effects are more pronounced after prolonged exposure to polyphenols. From this perspective, we have introduced a long-term treatment model that, in comparison to short-term curcumin effects, likely provides more accurate information on the epigenetic modifications occurring over time. There are several possible reasons for the success of such a model. First, it is generally believed that in contrast to synthetic DNMT inhibitors, dietary polyphenols in general have weaker effects in modulating DNA methylation. Hence, a long-term model probably better captures the cumulative efficacy of such compounds when studied for a longer time period of time, as such models more closely mimic the use of these diet-based botanicals for the chemoprevention of various cancers. Second, the constant and repeated exposure of cancer cells to such polyphenols can invoke cell-specific “epigenetic memory”, which could directly or indirectly contribute to the fine tuning of the “epigenetic code” over prolonged time periods and help contribute to their chemopreventive potential [42]. Therefore, our observations of a higher magnitude of methylation changes after long-term curcumin treatment in all 3 CRC cell lines is not entirely surprising, but a phenomenon that more adequately explains the anti-tumor epigenetic mechanisms modulated by curcumin.

In addition to hypermethylation of tumor suppressor genes, global hypomethylation is a frequent event during carcinogenesis [7], [8]. It has been previously hypothesized that hypomethylating agents may be a “double-edged sword”, which prevent hypermethylation of the tumor suppressor genes on one hand and increase chromosomal instability on the other [11], [41], [43]. While the risk of inducing chromosomal instability may be unavoidable during long-tern demethylation, genomic instability of this sort is not acceptable in a cancer prevention setting [7], [8], [44]. From this point of view, DNA methylation-modifying agents with little or no effect on global methylation, such as curcumin, may be a preferred clinical choice as well [7]. In this regard, our data provide several interesting observations for curcumin-mediated epigenetic changes in comparison to 5-aza-CdR. First, treatment with curcumin was associated with both hypo- and hypermethylation, while 5-aza-CdR exclusively induced global hypomethylation across the majority of the 27,000 CpG sites examined. Second, unlike 5-aza-CdR, curcumin-mediated changes were predominantly observed in partially-methylated CpG loci, suggesting a DNMT-independent mechanism. Third, while 5-aza-CdR has been linked with inadvertent activation of silenced oncogenes and induction of malignant properties [45]–[47], the anti-tumor effects of curcumin have thus far been related to its ability to block NF-*κ*B activation and its HAT-inhibitory effects [30].

In an effort to understand the mechanism of curcumin-mediated methylation alterations we have performed several experiments. First, we questioned if curcumin-induced methylation changes are influenced by DNMTs. We performed DNMT1 expression and activity analyses following treatment with curcumin, but we failed to observe any significant changes (data not shown), suggesting that modulating activity on DNA methylation by curcumin is independent of its direct effect on DNMTs. Next, we performed concurrent gene-expression microarray analysis in all cell lines after short- and long-term curcumin treatments Our analyses revealed that methylation profiles of short-term curcumin-treated cells were similar to control cells but significantly different from long-term treated cells (**Figure 7**), while the gene expression profiles in short- and long-term curcumin treated cells were remarkably similar hypothesizing that long-term curcumin treatment could impact DNA methylation through the regulation of gene expression. The ground-breaking work from the ENCODE project has recently highlighted the complexity of DNA methylation and its causation of gene silencing [48]. In particular, when the authors compared the transcription factor transcript levels in comparison to average methylation, a significant negative correlation between transcription factor expression and binding site methylation was observed for most (>70%) transcription factors. The authors strongly argued that methylation patterns paralleling cell-selective chromatin accessibility result from passive deposition after the vacation of transcription factors from regulatory DNA [48]. Indeed, curcumin is known to modulate multiple pathways, proteins and transcription factors in particular NF-κB and long term inactivation of those networks (**Figure 7**) could be responsible for modification of the cancer cell methylome.

Although our study demonstrates a systematic and compelling effect of curcumin on DNA methylation in CRC cells, it has some limitations. First, although we analyzed multiple cell lines, our data are based on *in vitro* observations and further animal and human studies are needed to validate these results. Second, another limitation of this work may be that although we have provided extensive data on DNA methylation, concomitant analyses of histone modifications were not performed and were beyond the scope of this project. Since curcumin is a known HAT inhibitor [15], we cannot exclude that the primary effect on HAT-modifications could also contribute to the changes in DNA methylation, as partially supported by our gene expression data and discussed above. Third, the cell death observed following low curcumin concentrations in the short-term studies reflects its acute effect only- and to observe significant changes in cellular methylome, cancer cells must undergo several cell divisions prior to appearance of visible methylation changes. However, as a long-term chemopreventative compound, it must prevent or trigger a cascade of molecular events (anti-inflammatory, anti-oxidant, and possibly epigenetic events, including DNA methylation changes) that directly or indirectly sustain its efficacy by interrupting or disrupting tumor cell growth and proliferation in the long-term. Such a long-term effect of curcumin in a clinical situation prevents formation of colorectal adenomas and cancers in humans, an effect that has already been studied in several clinical trials over the years. Although speculative, it is possible that curcumin-associated demethylation of genes in humans may in part be responsible for some of its chemopreventive and anti-cancer effects observed in already completed human clinical trials.

In summary, this study provides novel evidence that curcumin may exert its anti-cancer effect at least in part through epigenetic modulation of DNA methylation in CRC cells. We demonstrate that treatment of CRC cells with curcumin is associated with changes in DNA methylation and gene expression. Unlike 5-aza-CdR, curcumin induces methylation changes only in a subset of genes, which is distinct from the generalized genomic hypomethylation associated with increased genomic instability. Further studies are needed to evaluate the precise molecular mechanisms of curcumin-mediated methylation changes and estimate the potential of curcumin for inducing epigenetic modulations in CRC in various preclinical and clinical models.

## Supporting Information

Figure S1 Ingenuity Pathway Analysis (IPA) for the microarray gene expression pattern in curcumin treated HCT116 colon cancer cells demonstrates that NFkB pathway and its downstrean genes were significant targets of curcumin-induced methylation alterations in all cell lines. Red indicates hypomethylation/up-regulated and green indicates hypermethylation/down-regulated genes.
(TIF)

Table S1 Primer sequences for qMSP and quantitative bisulfite pyrosequencing analysis used in this study.
(DOC)
