# Supplementary figures and images for: Curcumin Modulates DNA Methylation in Colorectal Cancer Cells
Source: PLoS One. 2013 Feb 27;8(2):e57709. doi: 10.1371/journal.pone.0057709 (PMC3584082; doi:10.1371/journal.pone.0057709)

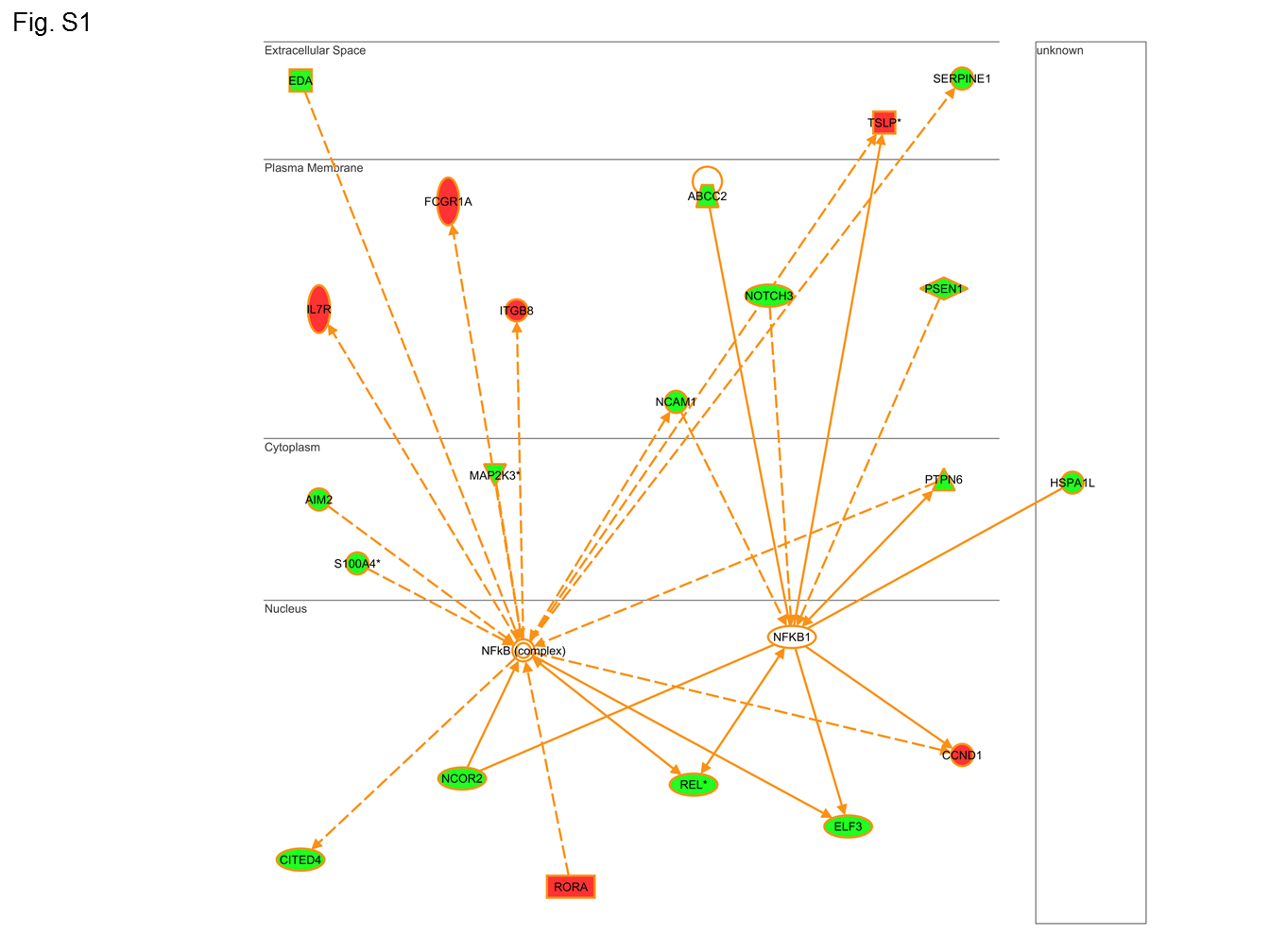

Supplement: Figure S1 — Ingenuity Pathway Analysis (IPA) for the microarray gene expression pattern in curcumin treated HCT116 colon cancer cells demonstrates that NFkB pathway and its downstrean genes were significant targets of curcumin-induced methylation alterations in all cell lines. Red indicates hypomethylation/up-regulated and green indicates hypermethylation/down-regulated genes. (TIF) [file pone.0057709.s001.tif]
