# Supplementary material for: Curcumin Modulates DNA Methylation in Colorectal Cancer Cells
Source: PLoS One. 2013 Feb 27;8(2):e57709. doi: 10.1371/journal.pone.0057709 (PMC3584082; doi:10.1371/journal.pone.0057709)
Supplement: Table S1 — Primer sequences for qMSP and quantitative bisulfite pyrosequencing analysis used in this study. (DOC) [file pone.0057709.s002.doc]

**Supplementary** **Table S1:** Primer sequences for various genes analyzed in this study

|  | ***Forward*** | ***Reverse*** | *PCR product size (bp)* |
| --- | --- | --- | --- |
| **qMSP** |  |  |  |
|  |  |  |  |
| GATA4 | GGAGTCGTTTTGAGGTGC | AAAAACAACGCGAACAAAT | 144 |
| KM-HN-1 | TATTCGGGTTTATCGTCGC | ACCCGATTACCTAACGACAAA | 100 |
| PTPRO | GCGTACGTTTTGTTGTTTTC | AAAACACAAAAAACGAAAACCG | 112 |
| WT1  CCND1  UCHL1  CCDC71 | GGGGTTCGTTGTAGAAGC  TGGGTCGTTATATTTTTGGC  Tcgtatttatttggtcgcgatc  GGTTTGGAGTAGTTATCGC | CTTTCGACAAAAAAACGACC  TCTTTATCGACCCCATCG  Ctataaaacgccgaccaaacg  ATACACTACGCTTCCGCT | 148  135  105  149 |
| **Pyrosequencing** |  |  |  |
|  |  |  |  |
| LINE-1 | TTTTGAGTTAGGTGTGGGATATA | 5’-biotin-AAAATCAAAAAATTCCCTTTC |  |
| LINE-1 pyro-seq. | AGTTAGGTGTGGGATATAGT |  |  |
